# Accentuate the Positive: Semantic Access in English Compounds

**DOI:** 10.3389/fpsyg.2013.00203

**Published:** 2013-04-24

**Authors:** Victor Kuperman

**Affiliations:** ^1^Department of Linguistics and Languages, McMaster UniversityHamilton, ON, Canada

**Keywords:** morphological processing, semantics, emotion, valence, lexical decision

## Abstract

The present study supplements research on semantic effects in word processing by focusing on the role that meanings of morphemes play in recognition of complex words. We present an overview of behavioral effects of six semantic properties characterizing the emotional and sensory connotations of English compounds and their morphemes, as well as their semantic richness. Semantics of compounds affected latencies to those compounds, and semantics of morphemes affected latencies to those morphemes presented as isolated words. Yet semantics of morphemes had little bearing on recognition of compounds, with the exception of longer recognition times for compounds with emotionally negative morphemes (e.g., seasick). We interpret the data as evidence against obligatory decomposition and dual-route accounts of morphological processing and in favor of the naive discriminative learning account that posits independent, morphologically unmediated, and simultaneous access to all meanings activated by orthographic cues in the visual input. We discuss selectivity and division of attention as driving forces in complex word recognition.

## Introduction

What role do morphemes play in visual recognition of complex words? This question has been on the forefront of research on morphological processing for about four decades and gave rise to a spectrum of processing theories (for recent reviews, see Amenta and Crepaldi, 2012; Diependaele et al., 2012). Under strong decompositional proposals (Taft, 2004; Rastle and Davis, 2008; among others), complex words (or words that appear complex) undergo obligatory decomposition into morphemes based solely on formal (orthographic and phonological) cues, after which meanings of morphemes are accessed and recombined into a unified representation of the entire complex word. Repair strategies, with concomitant direct access to the meaning of the entire complex word, are argued to exist for cases when such integration is impeded by morphological misparsing or by semantic opacity of the word (e.g., *corner*, *hogwash*) (Marslen-Wilson et al., 1994). Alternatively, dual- and multiple-route models propose that the meanings of both the complex word and its morphemes can be activated simultaneously: moreover, the processing preference for either the morphemic or the whole-word route is not categorical and can be biased by the formal properties of the complex word (e.g., Caramazza et al., 1988; Schreuder and Baayen, 1995; Kuperman et al., 2008). A radically different class of models does away with morphemes as an independent level of representation and argues for a learned mapping between co-activating formal and semantic units that is either direct (Baayen et al., 2011) or mediated by a layer of hidden units (Seidenberg and Gonnerman, 2000). The success of adjudicating between processing accounts critically depends on the ability to pin down semantic access to complex words or their morphemes in an easily measurable and unequivocal way. In this paper, we critically review the current practices of identifying semantic access and propose complementary diagnostic measures.

Perhaps the most relied-upon indices of access to the meanings of morphemes and the words which embed them are the effects of complex word frequency and of morpheme frequencies on (neuro-)behavioral correlates of word recognition. Lexical frequencies are either manipulated orthogonally through a factorial design, or are allowed to vary across words, in which case the variability is accounted for using regression methods. The underlying assumption is that whole-word or morpheme frequencies (e.g., frequency of *doghouse* or *dog*) can only influence dependent variables (e.g., a response latency to *doghouse*) in a reliable way if those words or their morphemes are accessed in the long-term lexical memory of the reader. A whole-word frequency effect is often taken as a sign of full-form access, while morpheme frequency effects are taken as a sign of morphemic access (for an alternative interpretation see e.g., Baayen et al., 2007). The same logic applies when morphological family size (the number of complex words sharing a morpheme) or related information-theoretical measures are used in diagnosing the magnitude and time-course of semantic access. Typically, higher-frequency compounds are processed faster, while compounds’ constituents that have higher frequencies or belong to larger families facilitate recognition of low-frequency compounds and impede recognition of higher-frequency compounds (see the target list of processing phenomena and respective references in Amenta and Crepaldi, 2012). To give only one example of the utility of this general approach, Baayen et al. (2007) demonstrated robust effects of whole-word frequency but only weak effects of stem frequency on lexical decision latencies to derived words across the entire frequency range. This finding served as a counter-argument to an influential proposal (Alegre and Gordon, 1999; Pinker, 1999) that low-frequency derived and inflected words are solely processed on the basis of their morphemes and do not form whole-word representations.

For the frequency of a complex word, a morpheme, or a set of morphological family members to be indicative of semantic access, one has to assume that these effects are semantic in nature. Indeed, word frequency has been argued to have a strong semantic component: high frequency words are semantically richer (have a large number of associations and synonyms, Balota et al., 2004), and cluster together with semantic variables as predictors of lexical decision latencies (Baayen et al., 2006). Yet, the effect of word frequency could also arise at the level of form: strings that are encountered more often and better entrenched in the mental lexicon are easier to recognize, even if only partial orthographic information is available (de Almeida and Libben, 2002). Also, a complex word’s frequency may be construed as the joint frequency of its morphological constituents (Baayen et al., 2007). The higher the frequency of a complex word in a language, the stronger the association between that word and its morphemes, and the more experience the reader has with integrating a given morpheme into that embedding word. If so, a high frequency compound may benefit more from identification of one of its constituents than a low-frequency compound on purely formal – orthographic or phonological – grounds. No access to semantics of either morpheme or their combination is required. Likewise, in processing accounts that eschew morphemes, a higher-frequency string of characters that is embedded in a complex word or that constitutes the entire complex word, may lead to an easier activation of that word without recourse to semantics.

A semantic component in the effects of morphological families on complex word recognition has been demonstrated by Moscoso del Prado Martín et al. (2005) who showed that semantically unrelated members of families (e.g., *deadline* in the right positional family of *line*) inhibit the processing of other family members, while related ones facilitate this processing; see also De Jong et al. (2000) and De Jong et al. (2002). Yet it is conceivable that effects of morphological families on reading behavior are also partly due to the experience of encountering and identifying strings (morphemes) that are often embedded in larger strings (complex words). Thus, there may be circumstances in which effects of morphological families arise without recourse to semantic properties of either morphemes or words, making difficult the determination of the time-course or the presence of semantic access.

The partial form-dependence of frequency effects discussed above is complemented in literature by more direct use of semantic properties. These include semantic transparency (e.g., gaged as a subjective rating of how predictable the meaning of a complex word (*department*) is given the meanings of its parts (*depart* and *ment*); e.g., Libben et al., 2003), semantic similarity (gauged via the Latent Semantic Analysis measure of semantic distance between complex words and constituents; e.g., Landauer and Dumais, 1997; Ji et al., 2011; Marelli and Luzzatti, 2012; Kuperman et al., submitted), and relational structure (gauged as a type and relative frequency of the specific conceptual association between constituents in a complex word, e.g., teacup is a cup FOR tea; see Gagné and Shoben, 1997; Pham and Baayen, in press). A large body of masked or unmasked priming lexical decision studies choose semantic transparency and similarity as their critical experimental manipulation. The frequent cross-linguistic observation that semantically related and unrelated prime-target pairs produce equivalent amounts of the priming benefit is interpreted as an index of the semantically blind nature of early decomposition of complex words into morphemes (Rastle et al., 2004; Rastle and Davis, 2008; see however Feldman et al., 2009). Whether or not effects of semantic transparency are present in word recognition in context has been in focus of several eye-tracking studies (Pollatsek and Hyönä, 2005; Juhasz, 2007; Frisson et al., 2008; Marelli and Luzzatti, 2012; Kuperman et al., submitted), and the results are mixed. What these semantic properties have in common is that they are *relational*, i.e., minimally require a simultaneous evaluation of two meanings: those of two constituents, or a constituent and a compound. (Considered in isolation, a word is neither transparent nor opaque.) The relational nature of these measures does not allow for an assessment of whether or when an individual “atomic” lexical meaning of a morpheme or a complex word is accessed. Also, as will become important below, relational measures operate on lexical denotations, i.e., dictionary meanings of words, rather than connotations, i.e., non-literal aspects of word meaning.

The present paper takes as the point of onset a long-standing observation that recognition of a word is not confined to the identification of its referent (or often, multiple referents). Rather it evokes a rich connotative semantic palette which encompasses, among others, both the word’s connectedness with words sharing semantic features (e.g., syno-, anto-, hypo- and hypernyms, or associates), the word’s embodiment in the physical world (e.g., concreteness and imageability), and the emotional state that the word elicits (e.g., positivity, arousal, danger, and usefulness), see reviews by Balota et al. (2006) and Lupker (2008). The presence or absence of connotative effects, which robustly accompany lexical access, would indicate whether (and potentially, when) the meaning of a specific lexical item (word or morpheme) is activated. For instance, in the obligatory decomposition model morphemes are segmented out of the complex words either on purely morpho-orthographic or morpho-semantic grounds (see Feldman et al., 2009 and Davis and Rastle, 2010 for discussion of conflicting positions): thus whether or not to expect the effect of relational semantic properties at this stage is open to debate, and this study is not designed to shed light on this topic. Meanings of morphemes are further accessed, at which point we expect atomic connotations of morphemes to produce effects, and then morphemic meanings are recombined into a compound’s meaning, at which point we expect relational properties assessing the similarity between those meanings to play a role. The end result of the obligatory decomposition account is access to the compound’s meaning, so we additionally expect connotations of that meaning to influence recognition behavior. In the dual-route model, one expects to see effects of semantic properties stemming both from morphemes and whole compounds, because both routes are potentially in use in recognition. Predictions for other accounts are worked out in the Discussion. The knowledge of when compounds or their morphemes are semantically activated is essential for specifying both the architecture of the mental lexicon and the relative order of cognitive operations that access the lexicon.

We aim to survey a range of lexical variables that are (a) unequivocally semantic and not reducible to form, (b) inherently present in any free morpheme or a morphologically complex word, and (c) atomic, i.e., do not require an evaluation against a similar value in another word to be effective as a diagnostic of lexical access. An ideal variable would satisfy conditions (a)-(c), be available for both the whole-word and its morphemes, and produce detectable effects, of both the whole-word and morphemic meanings, in tasks requiring word recognition. We expect this novel repertoire of atomic semantic properties applied to morphologically complex words to complement the current body of evidence obtained with distributional and relational measures.

Even though we focus on a single task, lexical decision, the present study also paves the way for expanding cross-task research on semantic processing. A recent line of inquiry (Pexman et al., 2002, 2008; Yap et al., 2011, 2012) examines effects of a broad range of semantic variables on tasks requiring word recognition (lexical decision, speeded naming, word identification in the progressive demasking paradigm, semantic classification, and others). Monitoring the presence of semantic effects across tasks points to the contribution of variables to task performance and the suitability of the task for tapping into semantic access. So far, simplex words have been in the center of all studies in semantic richness. Complex words, which are considered here, take this inquiry one step further, as they enable us to investigate multiple sources of semantic richness within a word, with meanings that vary between morphemes and the word as a whole (cf. dead, line, and deadline). We pursue these goals by considering semantic properties of a large set of English noun-noun unspaced compounds, along with lexical decision latencies to those compounds.

## Materials and Methods

### Variables

Our overview of lexical databases identified six candidate variables for diagnosing semantic access: subjective ratings of valence (emotional positivity), arousal, imageability, concreteness, the Sensory Experience Rating (henceforth, SER), and the body-object interaction (BOI) rating. Each of these variables has been shown to affect simplex word recognition. For instance, in the lexical decision task that is discussed here, words were processed faster if they were more positive or more arousing (with an unsettled debate on the interactions or polarity of these effects), more imageable, more concrete, were associated with a stronger sensory experience or with a referent that can be physically interacted with, or produced more associates in the free-association task (Estes and Adelman, 2008; Larsen et al., 2008; Kousta et al., 2009; Juhasz et al., 2011; Yap et al., 2012; Juhasz and Yap, 2013).

The selected semantic measures quantify how deeply it is embodied in the emotional or sensory (especially, visual) experience with the physical world. Two of the measures – *valence* and *arousal* – tap into the emotional connotations that the word is associated with. We used Warriner et al.’s (in press) emotional ratings that expand the Affective Norms for English Words (ANEW) database (Bradley and Lang, 1999) to about 14,000 words and implement the dimensional three-scale assessment of emotions as proposed in Osgood et al. (1957) theory of emotion (we only consider two, as dominance is highly correlated with valence). Valence, or emotional positivity, gages the amount of pleasantness or discomfort that a person feels when reading the word, and is measured on a scale from 1 (sad, unhappy) to 9 (happy). Words with extreme average valence ratings are *pedophile* (1.26) and *vacation* (8.53). Arousal assesses the level of excitement that raters associate with the read word, and is measured on a scale from 1 (calm) to 9 (excited). Words with extreme average arousal ratings are *grain* (1.6) and *insanity* (7.86). Four other measures – *imageability*, *concreteness*, the body-object interaction *BOI* rating, and the strength of sensory experience *SER* rating – assess the experiential link between the concept that the word’s referent represents and its embodiment in the physical world. *Imageability* evaluates, on a scale from 1 to 7, how easily the word elicits a “mental image (i.e., a mental picture or sound, or other sensory experience)” (Cortese and Fugett, 2004). Words with extreme imageability ratings are *is* (1.2) and *hammer* (7.0). Concreteness assesses, on a scale from 1 to 5, how easily the referent of the word can be seen, heard, felt, smelled, or tasted (Spreen and Schulz, 1966; Brysbaert et al., in preparation). Words with extreme average concreteness ratings are: *essentialness* (1.04) and *flashlight* (5.00). The body-object interaction rating evaluates, on a scale from 1 to 7, the ease with which a human body can manipulate the referent of the word in the physical world (Tillotson et al., 2008). Words with extreme average BOI ratings are *blah* (1.17) and *ball* (6.66). The strength of sensory experience ratings evaluate, on a scale from 1 to 7, the degree of sensory experience (an actual sensation like taste, touch, sight, sound or small) a word evokes (Juhasz et al., 2011). Words with extreme SER ratings are *a* (1.0) and *garlic* (6.56). The reader is referred to original papers for details on the formulation of task instructions and data collection, see Table 1 for references. Finally, we considered the number of associates in the free association task (*NoA*, Nelson et al., 2004) as a measure of the semantic connectedness of a word with concepts represented by other words (Duñabeitia et al., 2008; Rabovsky et al., 2012; Yap et al., 2012). NoA did not reach significance in any of the models fitted to RTs for stand-alone morphemes, and is not reported further. We opted out of using several other well-documented lexical properties either because they are – at least partly – form-related (age-of-acquisition, subjective frequency, subjective familiarity, contextual availability) or because their norms are available for only a small number of complex words: e.g., number of semantic features (McRae et al., 2005); mode of acquisition (Della Rosa et al., 2010), or meaningfulness (Chumbley and Balota, 1984).

**Table 1:** **Summary of datasets of semantic variables used in this study**.

Variable	Dataset size	Subset size
Valence*^a^*	13915	557
Arousal*^a^*	13915	557
Imageability*^b^*	5988	997
Concreteness*^c^*	300039	704
Sensory experience rating (SER)*^d^*	5857	998
Body-object interaction (BOI)*^e^*	1618	697

*The table reports (a) the size of the original dataset, and (b) the size of the subset of word triplets (compounds occurring in the CELEX database and their constituents) or word pairs (both constituents of compounds occurring in the CELEX database) that occur in the English Lexicon Project database, and (c) whether or not the subset included compound words*.

*^*a*^Warriner et al. (in press); *^b^*Cortese and Fugett (2004), Schock et al. (2012); *^c^*Brysbaert et al. (in preparation); *^d^*Juhasz et al. (2011), Juhasz and Yap (2013); *^e^* Tillotson et al. (2008)*.

The variables we selected encompass multiple aspects of word meaning, i.e., its emotional connotations, its embodiment in the physical world and its connectedness with other word meanings. Moreover, all these variables have been shown to affect simplex word recognition latencies and accuracy, see references for respective variables in this section. In contrast to relational measures like semantic transparency or similarity, values for these variables can, in principle, be independently estimated for both complex words (say, imageability of *doghouse* or *unable*) and each of their free morpheme constituents (imageability of *dog* and *house*, and of *able*). As argued above, whether or not these properties affect the response time to a word is a strong indication of semantic access to that word.

#### Control variables

To eliminate confounds with benchmark predictors of word recognition speed, we entered word length (in characters) as a control variable in our model. Furthermore, we included log-transformed frequencies of the compound and its left and right constituents (all based on the 16-million-token CELEX database, Baayen et al., 1995). The dependent variables were the item-average lexical decision latencies to existing compounds and constituents. Given the skewed nature of the response time distributions, we log-transformed all response times to approximate the normality of the distribution (the logarithmic transformation was indicated by the Box-Cox test, see Kliegl et al., 2010).

While there were no noticeable correlations between semantic properties of individual constituents, some values correlated between constituents and compounds: e.g., the valence of compounds correlated with the valence of either constituent at *r* = 0.3. To avoid the inaccuracy in the estimate of standard error which is typical in the presence of collinearity, we considered residuals of the regression model in which the influence of the left and right constituent’s valence were partialed out from the valence of the whole compound. The residual compound valence was strongly correlated with the original compound’s valence (*r* = 0.88) and had the additional benefit of being orthogonal to the valences of the constituents. The same procedure was applied to arousal and concreteness. As a result, there was no harmful collinearity in our sets of predictors (condition numbers that gage collinearity were κ < 10 in all our models; Baayen, 2008).

### Stimuli

The list of noun-noun compounds was compiled using the CELEX lexical database (Baayen et al., 1995). From each of the six datasets we extracted values for either pairs of words that are constituents in an English compound (*pine* and *apple*), or – if available – for word triplets, i.e., the two compound’s constituents and the whole compound (*pine*, *apple*, and *pineapple*). For instance, each of the 557 entries in the resulting subset of the valence dataset (Warriner et al., in press) corresponded to a triplet of ratings: positivity of the left constituent (year, 5.75), the right constituent (book, 7.05), and the compound (yearbook, 6.05), respectively. We further matched the resulting lists of words against lexical decision latencies to English compound words, as reported in the English Lexicon Project (Balota et al., 2007), see below. Sizes of the original datasets and the subsets of words overlapping with the English Lexicon Project data are reported in Table 1, and their distributional characteristics in Table 2. Valence and arousal ratings were available for a substantial number of compound words and their constituents in the original data sets (Warriner et al., in press). For several other lexical variables (imageability, concreteness, body-object interaction, and sensory experience rating) we collected additional ratings to all compound words that had ratings for both constituents in the original datasets. The Amazon Mechanical Turk web-based crowdsourcing platform was used for data collection, with native English-speakers residing in the US as participants: see Kuperman et al. (2012) for details of the method. Original instructions were used to collect imageability (Cortese and Fugett, 2004), concreteness (Gilhooly and Logie, 1980), body-object interaction (Tillotson et al., 2008), and sensory experience (Juhasz et al., 2011) ratings, with slight modifications pertaining to the online method of data collection. After removing responses outside of the respective rating scale, each word received an average of 18 ratings for each type of rating.

**Table 2:** **Descriptive statistics of semantic variables used in the study**.

Variable	Distribution
Valence of left	*N* = 308; range = 1.89–8.21; *M* = 5.57 (1.12)
Valence of right	*N* = 261; range = 1.62–7.78; *M* = 5.59 (1.02)
Valence of compound	*N* = 503; range = 1.79–8.14; *M* = 5.33 (1.14)
Arousal of left	*N* = 308; range = 2.05–7.74; *M* = 3.95 (0.95)
Arousal of right	*N* = 261; range = 2.21–7.74; *M* = 3.89 (0.91)
Arousal of compound	*N* = 503; range = 2.25–6.91; *M* = 4.07 (0.90)
Imageability of left	*N* = 470; range = 1.6–6.9; *M* = 5.69 (1.11)
Imageability of right	*N* = 390; range = 1.7–7.0; *M* = 5.36 (1.18)
Imageability of compound	*N* = 997; range = 1.16–6.90; *M* = 4.6 (1.32)
Concreteness of left	*N* = 678; range = 1.61–5.00; *M* = 4.44 (0.54)
Concreteness of right	*N* = 713; range = 2.13–5.00; *M* = 4.17 (0.66)
Concreteness of compound	*N* = 704; range = 1.68–5.00; *M* = 4.22 (0.56)
SER of left	*N* = 479; range = 1.20–6.33; *M* = 3.23 (0.96)
SER of right	*N* = 383; range = 1.18–6.33; *M* = 3.05 (0.87)
SER of compound	*N* = 776; range = 1.40–6.26; *M* = 3.37 (0.87)
BOI of left	*N* = 318; range = 1.37–6.66; *M* = 4.28 (1.39)
BOI of right	*N* = 262; range = 1.40–6.66; *M* = 4.27 (1.41)
BOI of compound	*N* = 696; range = 1.17–6.94; *M* = 4.33 (1.42)

**N* is the count of unique constituents or compounds for which lexical decision times and frequency counts are available. Numbers in parentheses report standard deviations*.

Table 2 additionally reports the descriptive statistics (range, mean, and standard deviation) for each semantic property that was considered for compounds and their constituents.

### Procedure and participants

The English Lexicon Project (Balota et al., 2007) provides behavioral data for 40,481 words and 40,481 non-words, collected from 816 participants in the lexical decision task and 444 participants in the naming task. All participants were drawn from undergraduate participant pools, across six universities. We used this pool to extract response times to both noun-noun compounds, and their constituent nouns as isolated words. We chose not to analyze naming latencies to complex words, as speeded naming has been repeatedly shown to be a more *shallow* task in that it does not implicate word semantics (cf. e.g., Yap et al., 2012, and references therein) and can be performed on a purely formal basis.

## Results

As the first step of analysis, we fitted linear regression models to lexical decision latencies for the compounds’ left constituents (e.g., *year* in *yearbook*) as isolated words (RT to *year*). Each model contained one of the semantic properties of the left constituent word (its valence, arousal, imageability, concreteness, or SER) as a critical predictor, and the length and log-transformed frequency of the left constituent as control covariates. Similar models were fitted to response latencies to the right constituent as a stand-alone word (*book*) with one of the semantic properties of that constituent set as a critical predictor and additional controls. These models aimed at testing whether the semantic properties of the compound’s constituents elicit effects on recognition of those constituents as isolated words. These effects would further serve as a baseline for assessing the impact of semantic properties on the recognition of those same simplex words when they are embedded as constituents in a compound, see below. Effects of constituents’ properties on lexical decision latencies to constituents as isolated words are summarized in Table 3, columns B and C.

**Table 3:** **Summary of regression models fitted to lexical decision latencies to compounds’ left constituents presented as isolated words (column B), compound’s right constituents presented as isolated words (column C) and compound words (column D)**.

A. Variable	B. RT to left	C. RT to right	D. RT to compound
Valence of left	$\hat{\beta }=-0.012,$ *SE* = 0.004*, p* = 0.003		$\hat{\beta }=-0.010,$ *SE* = 0.004*, p* = 0.009
Valence of right		$\hat{\beta }=-0.017,$ *SE* = 0.005*, p* = 0.001	$\hat{\beta }=-0.014,$ *SE* = 0.005*, p* = 0.002
Valence of compound			$\hat{\beta }=-0.018,$ *SE* = 0.005*, p* < 0.001
Arousal of left	ns		ns
Arousal of right		$\hat{\beta }=-0.012,$ *SE* = 0.006*, p* = 0.040	ns
Arousal of compound			ns
Imageability of left	$\hat{\beta }=-0.018,$ *SE* = 0.002*, p* < 0.001		ns
Imageability of right		$\hat{\beta }=-0.018,$ *SE* = 0.003*, p* < 0.001	ns
Imageability of compound			$\hat{\beta }=-0.009,$ *SE* = 0.003*, p* = 0.002
Concreteness of left	$\hat{\beta }=-0.014,$ *SE* = 0.004*, p* = 0.002		ns
Concreteness of right		$\hat{\beta }=-0.008,$ *SE* = 0.004*, p* = 0.044	ns
Concreteness of compound			$\hat{\beta }=-0.023,$ *SE* = 0.008*, p* = 0.008
SER of left	$\hat{\beta }=-0.021,$ *SE* = 0.002, *p* < 0.001		ns
SER of right		$\hat{\beta }=-0.021,$ *SE* = 0.003, *p* < 0.001	ns
SER of compound			$\hat{\beta }=-0.024,$ *SE* = 0.005, *p* < 0.001
BOI of left	$\hat{\beta }=-0.007,$ *SE* = 0.003, *p* = 0.012		ns
BOI of right		$\hat{\beta }=-0.008,$ *SE* = 0.003, *p* = 0.008	ns
BOI of compound			ns

*Column A lists critical predictors in the models. Estimated regression coefficients, standard errors and p-values are reported for all models in which critical predictors reached significance at the 0.05 level*.

All semantic properties of the right constituents were significant codeterminers of lexical decision latencies to words occurring as right constituents in the respective selections of compounds. Words elicited shorter response times if they carried a more positive or arousing meaning, were more imageable, concrete, associated with a stronger sensory experience or had a physically accessible referent. Same directions of effects were observed in our analysis of the latencies to left constituents as isolated words: arousal did not reach statistical significance at the 0.05 level. The directions of all effects converged perfectly with prior reports (see references in the Critical Variables section). The null effect of arousal of the left constituent word (e.g., *dog* in *doghouse*) on the lexical decision RT to this word (*dog*) is not surprising: arousal has been previously shown to affect lexical decision latencies only at some levels of the word’s valence (on the interaction of valence and arousal, see e.g., Estes and Adelman, 2008; Larsen et al., 2008; Kousta et al., 2009).

In the second step of our analysis, linear regression models were fitted to log-transformed average lexical decision latencies to compound words (*yearbook*). Each model included word length as well as compound and constituent frequencies as control covariates. Each model included also, as critical predictors, the ratings of one of the critical semantic properties (valence, arousal, imageability, concreteness, or SER) for the left and right constituents and, where applicable, for the entire compound, see Table 1. The effects of all semantic properties on lexical decision latencies to compounds are summarized in Table 3, column D.

### Valence

Valences of compounds and their constituents showed independent effects on lexical decision latencies. Both residual compound valence [$\hat{\beta }\;=\;-$0.018, SE = 0.005, p < 0.001], the valence of the left constituent [$\hat{\beta }\;=\;-$0.010, SE = 0.004, p = 0.009] and that of the right constituent [$\hat{\beta }\;=\;-$0.014, SE = 0.005, p = 0.002] affected log lexical decision latencies in such a way that a more emotionally negative compound or constituent led to slower responses. This is in line with the observation that Estes and Adelman (2008) made for a smaller subset of words (A follow-up analysis tested potential non-linearity of the valence effect and found no support for it: this runs counter to the observation of Kousta et al. (2009) that the speed-up in processing is characteristic of all non-neutral (positive or negative) words.). Moreover, a baseline model with word length and log-transformed compound and constituent frequencies explained 15.8% of unique variance in the data. A model with residual compound valence as an additional predictor explained 18.0%, while a model with all three valence predictors explained 20.2% of unique variance. Both increments in the amount of explained variance were highly significant (*ps* < 0.01). The joint consideration of baseline effects reported in columns B and C of Table 3 and current models (column D) reveal that the effects of constituent valences on recognition times retain their direction (negative) and magnitude (reflected in similar regression coefficients). This occurred even when constituents are not presented as isolated words but are orthographically embedded in a compound.

### Other variables

The impact of the remainder of the candidate variables can be summarized quite succinctly (see Table 3, column D). The speed of compound recognition was affected by semantic properties of compounds, but not by semantic properties of their constituents. Relatively imageable, concrete, and tangible compounds were processed faster: no reliable effect of arousal or BOI was observed. Yet, neither the arousal ratings of the constituents or the compounds, nor the imageability, concreteness, BOI, or SER ratings of constituents showed any reliable effects (at the 5% level of significance) on lexical decision latencies to English compounds. The consideration of non-linearity of effects, individual variability in sensitivity to semantic effects, and the interactions between semantic variables (e.g., the valence by arousal interaction) did not reveal any significant effects either. The null effects of all semantic properties of constituents (except valence) are in stark contrast with the significant impact that those same properties have on reaction times to those same constituent morphemes presented as isolated words, see columns B and C of Table 3.

## General Discussion

The present study set out to identify semantic lexical properties that influence word recognition behavior and represent inherent *atomic* properties of a stand-alone word (e.g., a word’s imageability) rather than *relational* properties of a word pair (semantic similarity). Six candidate properties were selected on the basis of their availability for a large number of English compounds and their constituents: emotional valence and arousal, imageability, concreteness, body-object interaction rating, and sensory experience rating. Values of these properties were obtained for large samples of English noun-noun compounds and for nouns that serve as constituents in those compounds. The values were further correlated with lexical decision latencies to compound words and their constituents presented in isolation. Our data demonstrate that atomic semantic properties of a word influence the ease of recognizing that word, regardless of whether the word is structurally simplex (*flash*) or not (*flashlight*), see Table 3. More specifically, recognition of a word not only comes with access to its denotation (dictionary meaning) which ensures success of reading comprehension in proficient readers, but also activation of such connotations as the emotional (valence) and sensory experience associated with the word’s referent (see references in the Introduction). Words with referents that are more positive, imageable, concrete, easy to manipulate in the physical world or evoke a stronger sensation are processed faster. However, when a simplex word is embedded in a compound as a morphological constituent, its semantics (except for its valence) loses influence on how this complex word is processed by the reader. The consistent null effects of morphemic semantics on compound recognition are not due to the insufficient statistical power, as the same samples of simplex words elicit equally consistent and reliable semantic effects when predicting latencies to morphemes presented as isolated words. If this were the entire body of evidence at our disposal, we would need to conclude that morphemes are not semantically accessed during compound recognition, and the only behaviorally detectable evaluation of meaning occurs at the level of the whole-word.

This preliminary conclusion needs to be qualified in light of widely reported behavioral effects of relational lexical properties, i.e., those that implicate semantics of lexical pairs. For instance, the Latent Semantic Analysis scores that estimate semantic similarity between the left constituent and the compound (*stop* vs. *stopwatch*) correlate with lexical decision latencies to 652 compound words in the English Lexicon Project at r = −0.143, while the right-whole similarity (*watch* vs. *stopwatch*) correlated with latencies at r = −0.128 (both p-values < 0.01; Kuperman et al., submitted), see also Järvikivi and Pyykkönen, 2011; Ji et al., 2011; Juhasz, 2007; Marelli and Luzzatti, 2012. Similarly, the availability or frequency of the conceptual relation between a compound’s constituents (armchair: chair WITH arms) has been shown to affect lexical decision latencies (Gagné and Spalding, 2004). These findings suggest that meanings of constituents get accessed in the process of word recognition, but only in the context of the compound’s meaning. More specifically, meanings of constituents appear to be weighed against the meaning of the compound either for similarity (how similar is boat to boathouse?), conceptual relation (how are arm and chair related in armchair?), or another kind of semantic associations.

Taken jointly, our findings suggest that meanings of compounds are accessed independently of those of morphemes, and the interpretation of morphemic meanings is constrained by the compound’s meaning. Three pieces of evidence support our claim. First, virtually none of the connotative atomic properties of a morpheme’s meaning was found to influence the recognition of complex words (an important exception of morphemic valence is discussed below). If morphemes were activated independently, we would expect to observe the same hallmark gamut of semantic effects (e.g., of valence, imageability, concreteness, SER) that accompanies access to those morphemes recognized as isolated words. Second, meanings of morphemes only play a role when compared against meanings of entire compounds as gauged by relational measures of semantic association: exclusively the denotations, and not connotations, of morphemes are implicated in those comparisons. Third, semantic access to compounds (e.g., flashlight) is as rich as that in simplex words presented in isolation, in that it evokes both the word’s denotation (a small battery-operated portable electric light, as defined in Merriam-Webster’s online dictionary, http://www.merriam-webster.com) and the degree of the word’s emotional and sensory embodiment in the physical world (a high valence, high imageability, high concreteness, high body-object interaction index, and high sensory experience rating). Meanings of embedded morphemes do not appear to modulate lexical access to compound words: in a series of models (not shown), we verified that word recognition behavior is not predicted by the similarity between the connotative atomic semantic properties of the constituents and those of compounds. It may be true that “the co-activation of many meanings provides evidence for lexicality that speeds lexical decision latencies” as stipulated by the multiple read-out model (Grainger and Jacobs, 1996), but not every aspect of meaning is activated, nor does it necessarily affect the ease of recognition, as far as embedded morphemes are concerned.

These observations run counter to any account that advocates access to meanings of morphemes as the initial, or even a substantial, step of arriving at a compound’s meaning. More pointedly, it is problematic for the obligatory decomposition account, which proposes – for most or all words – an independent activation of the meanings of morphemes and their recombination into a unified word meaning (e.g. Rastle et al., 2004; Taft, 2004; Rastle and Davis, 2008). It is also problematic for dual- and multiple-route models, which treat access to morphemic meanings, and their subsequent integration as a much-used route to recognizing a complex word (Caramazza et al., 1988; Baayen et al., 1997; Kuperman et al., 2008, 2009). At face value, the independent activation of compounds with a subsequent semantic activation of morphemes is compatible with the supralexical model of morphological processing by Giraudo and Grainger (2001), in which the orthographic cues of the complex word first provide access to that word’s meaning, then the activation trickles down to the word’s morphemes and morphemes send back the activation to all words that the morphemes are compatible with. Yet, since this account presupposes the full unconstrained activation of morphemes, even if after access to compounds, it is incompatible with the observation that morphemes are accessed only for some, denotational, aspects of their meaning and only in the context of the compound’s meaning.

We argue that the present pattern of results is consistent with the premises of the naive discriminative learning account of morphological processing (Baayen et al., 2011; Pham and Baayen, in press). On this approach, orthographic (and phonological) cues are directly mapped onto meanings, without the mediation of morphemes as a representational level. The weights of orthographic-semantic connections are a result of statistical learning obtained from experience with recognizing words in context. The model proposes that orthographic cues simultaneously activate a variety of meanings, including the meaning of the complex word, meanings of its morphemes, and meanings of non-morphemic strings that constitute lexical units (Bowers et al., 2005), e.g., shoestring, shoe, string, hoe, and ring. Pham and Baayen (in press) further suggest that selective attention is directed toward the meaning of the entire word (shoestring), with only marginal attentional resources allocated to other co-activated meanings. This contrasts with staged architectures of morphological processing, in which the compound meaning is singled out from competitors through the processes of interactive activation and inhibition of hierarchically organized units (morphemes and complex words).

To reiterate, processing efficiency demands that a preferred strategy is to direct the spotlight of selective attention to the meaning of the entire (simplex or complex) word, rather than its sublexical units. Several reasons speak in favor of this claim. First, the goal of reading, and of word recognition as its component, is to arrive at the whole-word’s meaning, so giving a privileged attentional status to this meaning, rather than dividing it over multiple entities, contributes to processing efficiency.

Second, focusing on meanings of morphemes into which a complex word can be decomposed is inefficient, because no compound meaning is truly compositional and even highly transparent compounds, e.g., *boathouse*, are not mere sums of the meanings of their constituents (Kuperman et al., submitted; Pham and Baayen, in press). For example, the Merriam-Webster dictionary provides the following two definitions for the word *boat*: (1a) a small vessel for travel on water, (b) ship; (2) a boat-shaped container, utensil, or device (e.g., a gravy boat, a laboratory boat). Nine definitions are available for the word *house*, ranging from “a building that serves as living quarters for one or a few families” to “the circular area 12 feet in diameter surrounding the tee and within which a curling stone must rest in order to count.” Semantic ambiguity abundant in the denotation of either constituent as a stand-alone word is only resolved when those constituents are pitted against each other in a compound: the combinatorial space of 2 × 9 = 18 possible meanings is reduced to one meaning, *a building to house and protect boats* rather than, say, *a legislative assembly of specifically shaped utensils*. Recombination of meanings obtained as a result of decomposition is then inefficient as it does not generally afford the intended idiosyncratic meaning of the compound. In fact, Kuperman et al. interpret effects of relational properties like semantic transparency or conceptual relations as corrective measures that aim to abate the consequences of the accidental activation of morphemic meanings, which adds undesired ambiguity about the compound meaning. Despite its inefficiency, an increase in morphemic activation may take place in compounds that require more than one eye fixation due to their length (leading to a recognition advantage of word parts rather than the whole), compounds that are spaced or hyphenated, or indeed compounds whose morphemes automatically capture the reader’s attention (see below), see Kuperman et al. (submitted).

Third, attending to the meanings of morphemes may be particularly harmful in the lexical decision task, the task under consideration. Most lexical decision experiments, including the English Lexicon Project megastudy used here, create non-existing equivalents of words by substituting several letters in *only one* of constituents (e.g., *jailbord* or *pootstool*), see Keuleers and Brysbaert (2010) for criteria of non-word generation. This implies that semantic properties of any single constituent do not discriminate between a word and a non-word. Assessing atomic semantic properties like valence, imageability, or concreteness of a constituent is thus inefficient in that it does not guarantee the lexicality of the string under recognition. Importantly, relational semantic properties fare better in this regard. Namely, a success or failure in evaluating the association between the meanings in a pair of linguistic units correlates perfectly with whether or not both units are lexically meaningful (e.g., no meaning is associated with *poot* in the example above, and so semantic transparency of *pootstool* cannot be evaluated).

To sum up, selective attention to compound meaning is preferred on the grounds of processing efficiency over attention divided over a gamut of co-activated meanings, much like a situation of a “car driver on a busy street focusing on a traffic light: only one object from a rich array of objects in the visual scene is attended” (Pham and Baayen, in press). If attentional focus on whole-words, including compounds, is indeed the driving force of word processing, we expect data to support the following two predictions. Recognizing whole-words, as compared to embedded words, would give rise to a richer perceptual experience, which would transcend the boundaries of the literal meanings: this prediction is borne out since imageability, concreteness, and other sensory characteristics of the word’s referent only affect one’s behavior when the word is not embedded in another word, and are similarly influential whether or not the recognized word was complex.

The second prediction posits an even stronger test of the attentional account of simultaneous semantic processing. Semantic properties that are known to automatically capture attention, and only those properties, are expected to cause the attention to divide between compounds and morphemes which would result in an effortful compound recognition. To stick to the metaphor, the flickering red of a traffic light may draw the driver’s attention and cause a slower response to the change from a red light to a green one. The effects of valence on behavioral latencies demonstrate that this prediction is borne out in our data too. Emotionally negative (simplex and complex) whole-words are recognized slower; moreover, compounds with emotionally negative morphemes show longer latencies as well. We propose that the privileged status of morphemic valence in complex word recognition stems from *automatic vigilance* to emotional information as an inherent property of human cognition (White, 1996). Information about entities that are harmful and threatening or, alternatively, entities that are beneficial and rewarding is obviously essential for one’s survival, as it triggers approach or avoidance behaviors in response to a stimulus (Mathews and Mackintosh, 1998; Wurm, 2007). Emotionally negative objects, including words, have been shown to capture covert attention automatically (cf. Stormark et al., 1995; Chen and Bargh, 1999). Delayed disengagement of attention from negative stimuli causes slower responses to negative words in both tasks that do not require word recognition (e.g., the emotional Stroop test, Pratto and John, 1991), and tasks requiring word recognition (Estes and Adelman, 2008; Scott et al., 2009, 2012; Kryuchkova et al., 2012; see however Larsen et al., 2008; Kousta et al., 2009). In the present data, emotional negativity captures the reader’s attention even if negativity is conveyed by the string embedded in a larger string (e.g., sick in seasick). Importantly, this happens to the detriment of processing. The inefficiency caused by a stronger semantic activation of relatively negative morphemes and reflected in longer response times suggests that valence, unlike other atomic semantic properties, cannot be turned off in a strategic way. The discrepancy between valence and other semantic properties follows from the premises of attentional focus on a compound: conversely, in models positing a stage with undivided attention to morphemes, vigilance is expected to be no more and no less salient for the reader than imageability, concreteness, or other connotative properties of the morpheme’s meaning.

In summary, the present study tests the impact that inherent connotative meanings of English compounds and their morphological constituents have on the visual processing of compounds. A key finding is that the observed patterns are most consistent with the proposal of the naive discriminative learning model (Baayen et al., 2011; Kuperman et al., submitted): a plethora of meanings receive unmediated, simultaneous, and fast activation from the orthographic cues, and selective attention to the activated meanings of whole-words (simplex or complex) is not constrained by meanings of embedded strings (morphemes). Conversely, the semantics of words embedded as morphemes appears to be constrained for interpretation by the context of the compound’s meaning, which is inconsistent with proposals advocating independent access to morphemic meanings and their further recombination into a semantic representation of a compound. Another key finding of the paper is the privileged role of emotional positivity (valence), which was the only semantic property of morphemes to have behavioral consequences on compound processing due to its attention-capturing ability. The role of valence is unquestionably strong in the visual comprehension of morphologically simplex words (see review of Citron, 2012). This study points to a similar role of valence in complex words, which embed several strings, several meanings and thus several dissimilar sources of either positive or negative information [compare a high valence rating of *sea* (6.56), a low rating of *sick* (2.29), and an even lower rating of *seasick* (1.89)]. We believe that the automaticity of attention-capture by the valence of morphological constituents can be used to accurately chart the time-course of semantic activation and resolve the long-standing debate on the amount of temporal overlap between the activation of form and meaning in morphological processing (cf. Amenta and Crepaldi, 2012 for review). Likewise, this study justifies the inclusion of connotative semantic effects like valence into models of both simplex and complex word recognition.

## Conflict of Interest Statement

The authors declare that the research was conducted in the absence of any commercial or financial relationships that could be construed as a potential conflict of interest.
